# Design, Synthesis and Biological Evaluation of Novel Piperazine Derivatives as CCR5 Antagonists

**DOI:** 10.1371/journal.pone.0053636

**Published:** 2013-01-07

**Authors:** Tao Liu, Zhiyong Weng, Xiaowu Dong, Linjie Chen, Ling Ma, Shan Cen, Naiming Zhou, Yongzhou Hu

**Affiliations:** 1 Zhejiang University-École Normale Supérieure Joint Laboratory of Medicinal Chemistry, College of Pharmaceutical Sciences, Zhejiang University, Hangzhou, China; 2 Institute of Biochemistry, College of Life Sciences, Zhejiang University, Hangzhou, China; 3 Insititute of Medicinal Biotechnology, Chinese Academy of Medical Sciences, Beijing, China; 4 Peking Union Medical College, Beijing, China; McGill University AIDS Centre, Canada

## Abstract

By using a fragment-assembly strategy and bioisosteric-replacement principle, a series of novel piperazine derivatives were designed, synthesized, and evaluated for their cellular target-effector fusion activities and in vitro antiviral activities against HIV-1. Preliminary structure-activity relationships (SARs) of target compounds were concluded in this study, and five compounds were found to exhibited medium to potent CCR5 fusion activities with IC_50_ values in low micromolar level. Among evaluated compounds, **23 h** was found to be a CCR5 antagonist with an IC_50_ value of 6.29 µM and an anti-HIV-1 inhibitor with an IC_50_ value of 0.44 µM.

## Introduction

AIDS, one of the leading threats for human health worldwide, is a disease of the human immune system caused by the human immunodeficiency virus (HIV). Although the highly active antiretroviral therapy (HAART) is an available option for AIDS treatment, many patients are suffered from incomplete efficacy, severe toxicity, and the eventual emergence of resistance [1]. Therefore, the development of potent antiretroviral agents with novel mechanism of action is of great interests in the field of medicinal chemistry and drug discovery.

**Figure 1:**
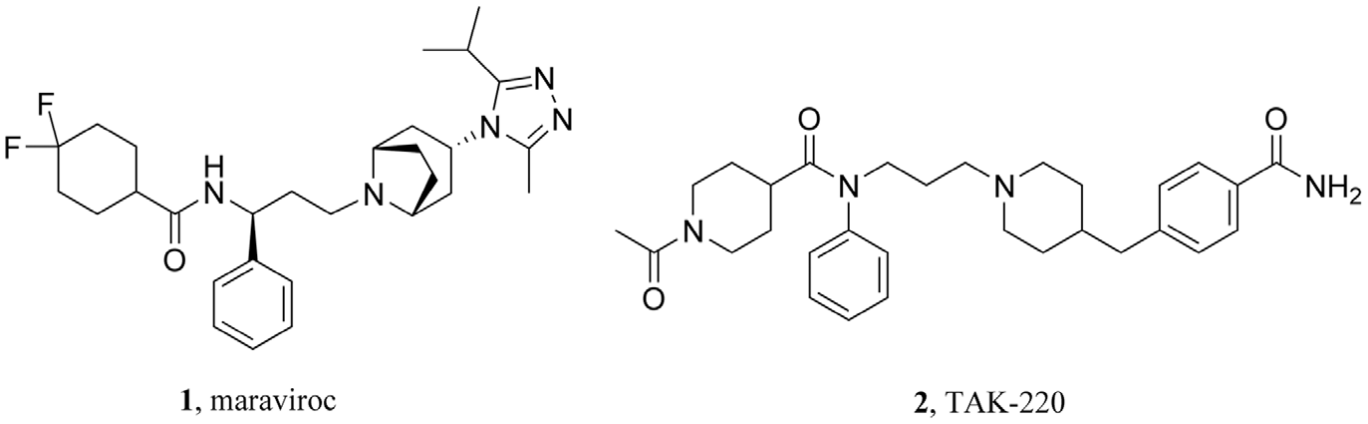
Representative structures of small-molecule CCR5 antagonists.

C-C Chemokine receptor 5 (CCR5), a G protein-coupled receptor (GPCR) for the β-chemokines MIP-1α, MIP-1β, and RANTES [2] and a primary co-receptor with CD4 for macrophage-tropic (M-tropic or R5) HIV-1 viruses [3] has been identified as a new target for HIV-1 epidemic prevention and treatment. Efforts devoted to the development of CCR5 antagonists have resulted in the discovery of the first marketed small-molecule inhibitor against CCR5, maraviroc (UK-427,857, **1**, Figure 1) [4]. Besides, several other promising molecules are currently under clinical trials as potential anti-HIV agents, such as the Takeda disclosed compound TAK-220 (**2**) [5], [6].

**Figure 2:**
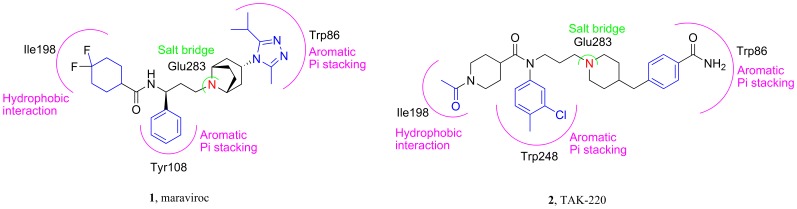
The binding models for maraviroc and TAK-220.

**Figure 3:**
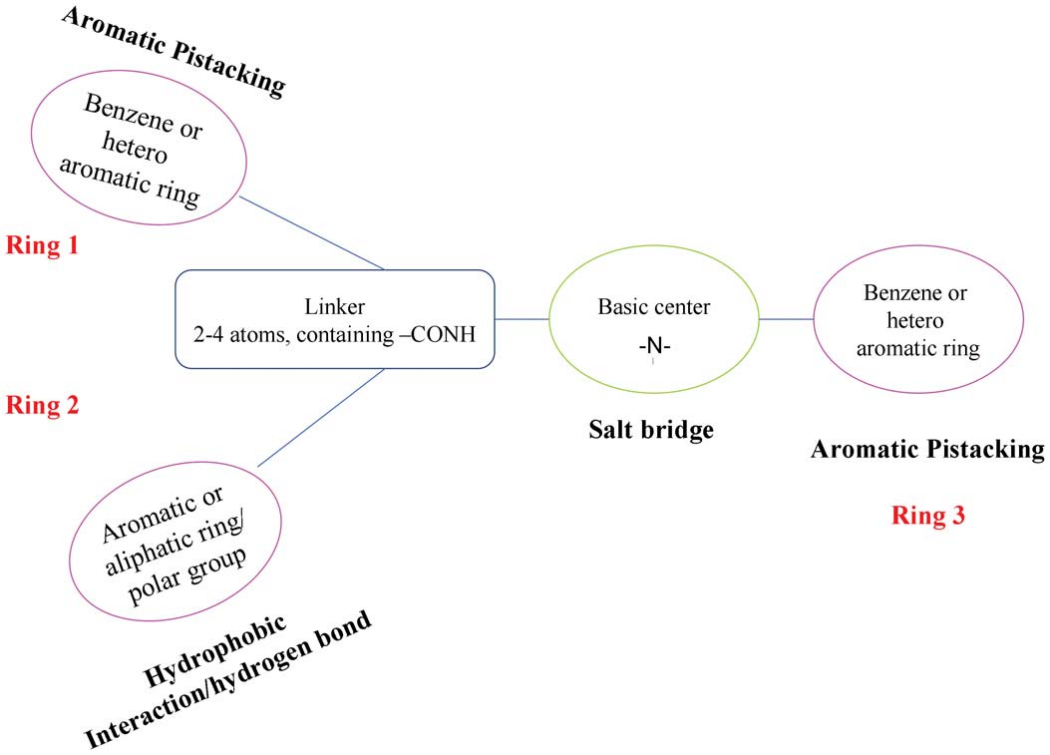
Proposed ‘Y shape’pharmacophore model of CCR5 antagonists.

Although reported CCR5 antagonists are of different structures, the presence of one basic nitrogen center that tends to form strong salt-bridge interaction with the Glu283 residue of CCR5 receptor was found to be one of the most important features for CCR5 antagonists. A hydrophobic interaction involving the Ile198 residue was found for both maraviroc and TAK-220, together with a T-shaped π-π stacking interaction involving the Trp86 residue (Figure 2) [7]. Thus, a ‘Y shape’ pharmacophore model that contains one basic center, three hydrophobic domains, and an amide linker (Figure 3) was proposed in this study.

## Results and Discussion

### Chemistry

**Figure 4:**
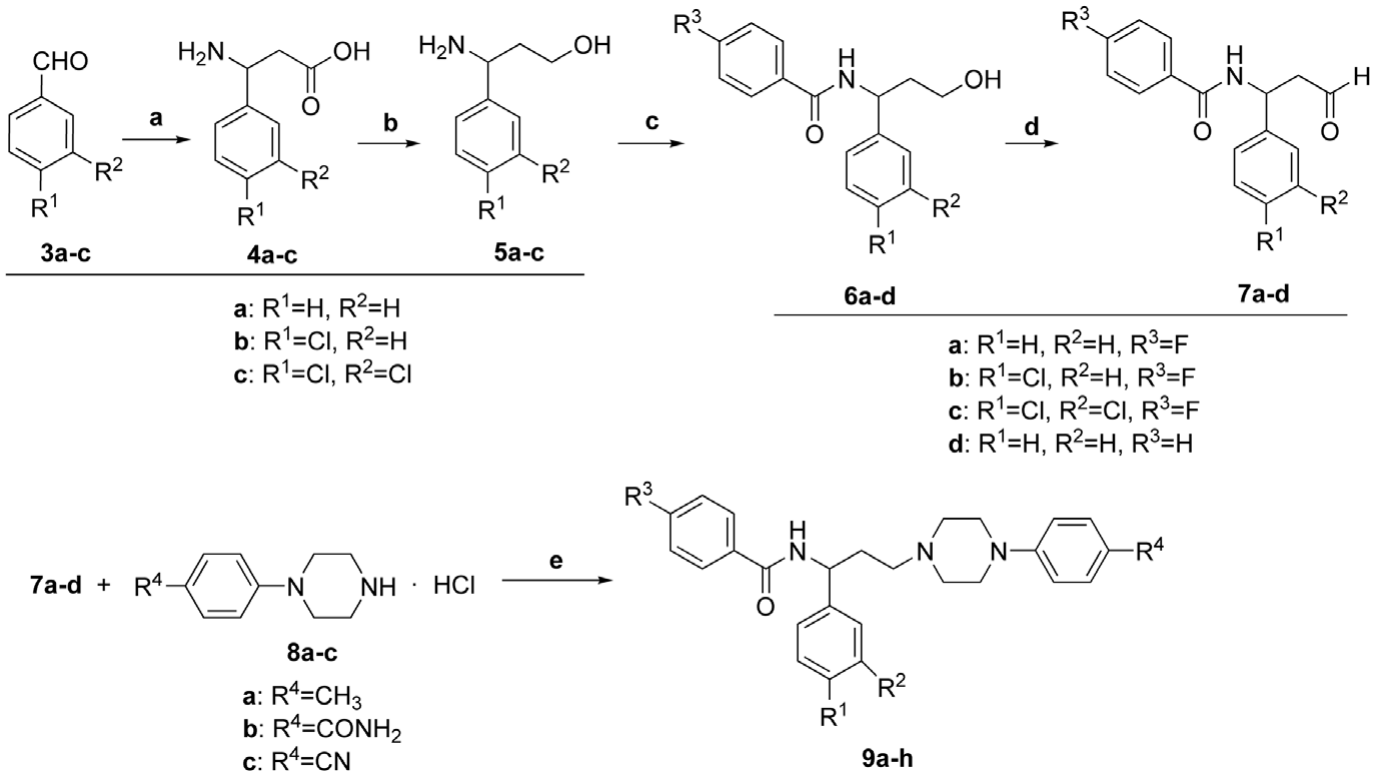
Synthesis of target piperazine derivatives 9a–h. Reagents and conditions: a) CH_3_COONH_4_, CH_2_(COOH)_2_, C_2_H_5_OH, reflux, 12****h; b) LiAlH_4_, THF, 65°C, 3****h; c) corresponding benzoyl chlorides, Et_3_N, CH_2_Cl_2_, 0°C, 4****h; d) (COCl)_2_, DMSO, CH_2_Cl_2_, −78°C, 2****h; e) NaBH(OAc)_3_, Et_3_N, CH_2_Cl_2_, rt, 8****h.

Target compounds listed in Tables **1** **,** **2** **, and** **3** were synthesized as outlined in Figure **4** **,** **5** **, and** **6**. As shown in Figure **4**, benzaldehydes **3a–c** were reacted with malonic acid and ammonium acetate to give β**-**amino acids **4a–c**, which was reduced to γ-amino alcohols **5a–c** with the presence of LiAlH_4_ [11]. Acylation of **5a–c** with corresponding benzoyl chlorides afforded amides **6a–d**. Compounds **6a–d** were then subjected to a Swern oxidation to give aldehydes **7a–d**, whose following reductive amination with substituted phenylpiperazine hydrochlorides **8a–c** [12] afforded target compounds **9a–h**.

**Figure 5:**
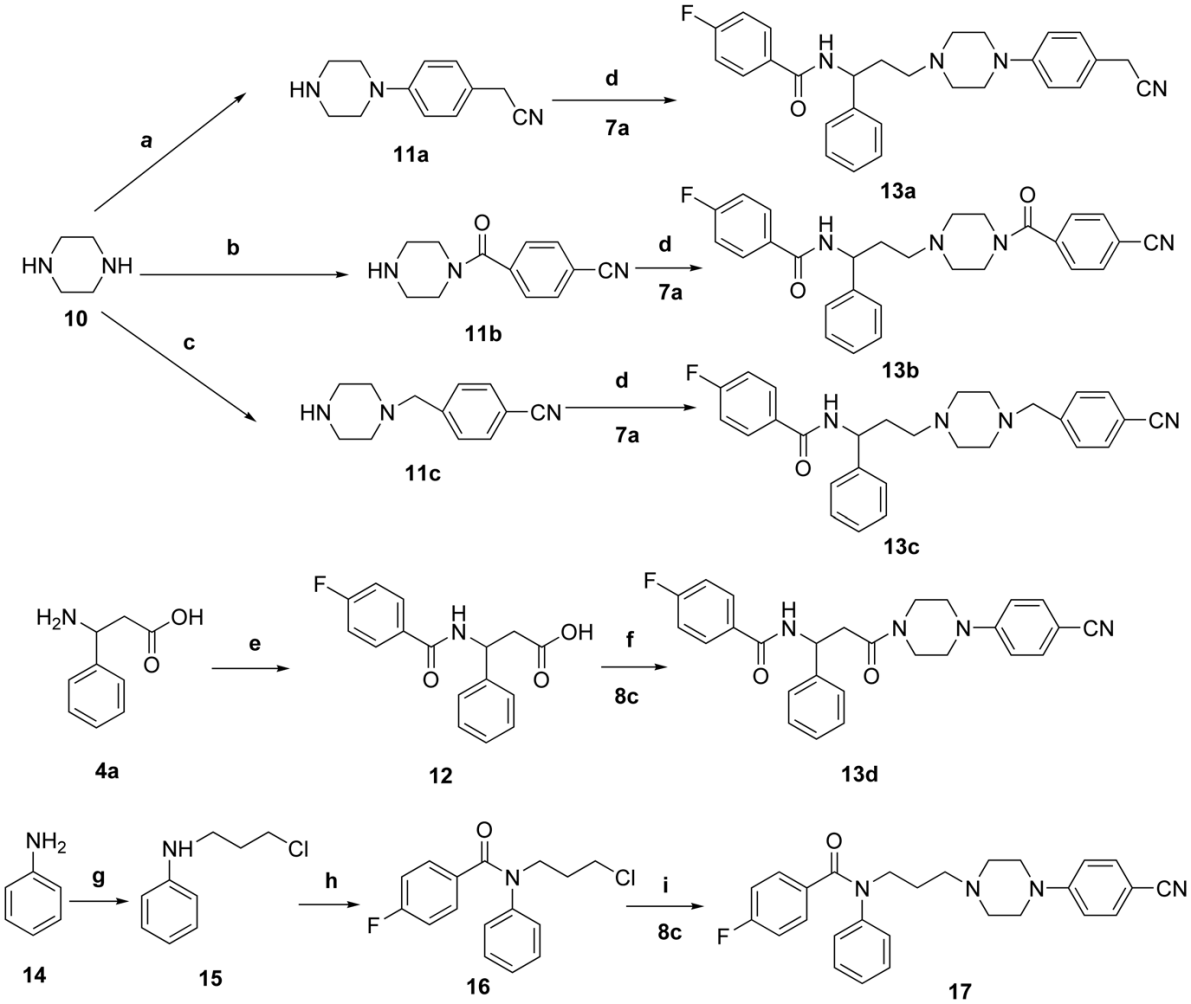
Synthesis of target piperazine derivatives 13a–d and 17. Reagents and conditions: a) 2-(4-chlorophenyl)acetonitrile, K_2_CO_3_, DMSO, reflux, 18****h; b) 4-cyanobenzoyl chloride, Et_3_N, CH_2_Cl_2_, rt, 3****h; c) 4-(chloromethyl)benzonitrile, THF, reflux, 2.5****h; d) NaBH(OAc)_3_, CH_2_Cl_2_, rt, 12****h; e) 4-fluorobenzoyl chloride, Et_3_N, CH_2_Cl_2_, rt, 3****h; f) EDC.HCl, CH_2_Cl_2_, rt, 8****h; g) 1-bromo-3-chloropropane, KI, CH_3_CN, MWI, 15****min; h) 4-fluorobenzoyl chloride, Et_3_N, CH_2_Cl_2_, 0°C, 5****h; i) KI, K_2_CO_3_, CH_3_CN, reflux, 24****h.

**Figure 6:**
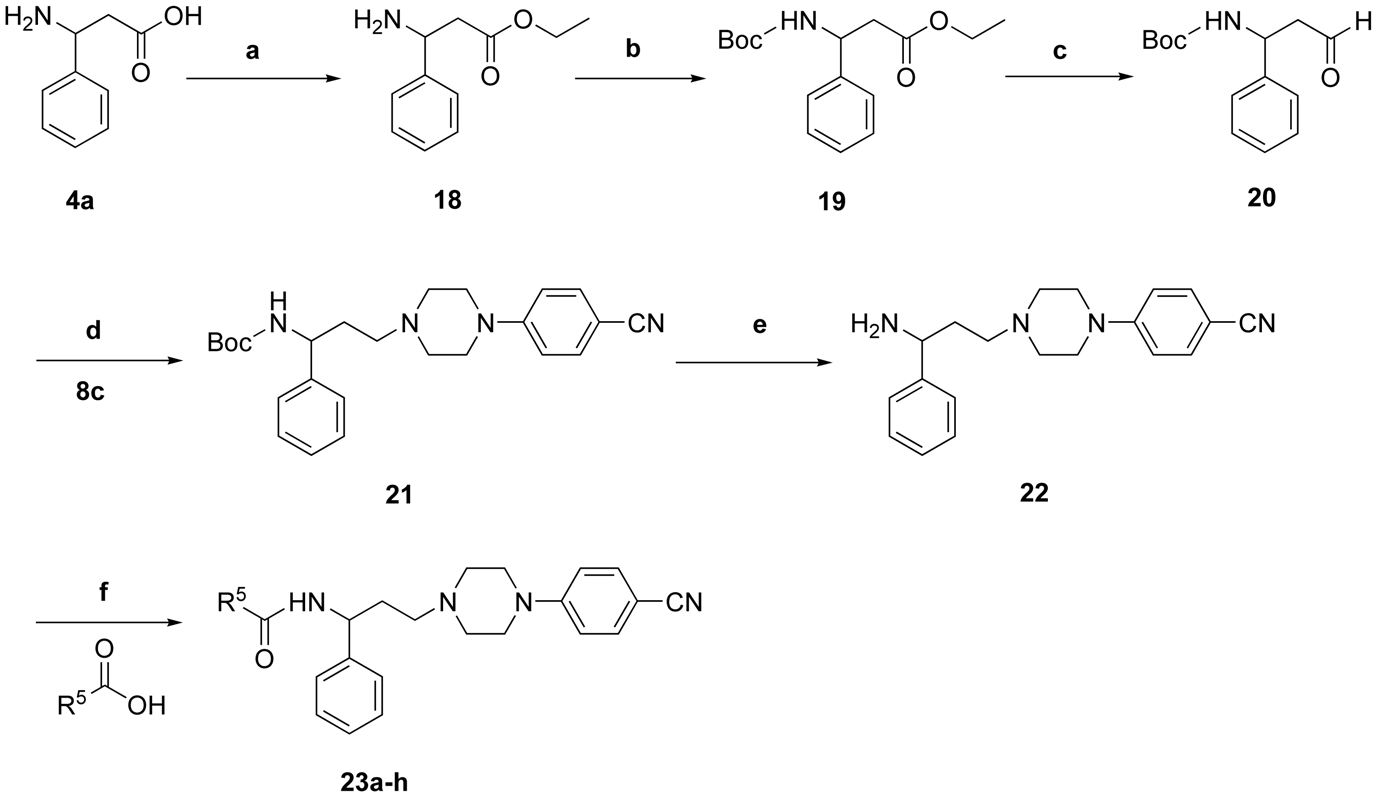
Synthesis of target piperazine derivatives 23a–h. Reagents and conditions: a) SOCl_2_, EtOH, 0°C, 0.5****h, rt, 3****h, reflux, 1****h; b) (Boc)_2_O, NaOH, H_2_O, Dioxane, rt, 12****h; c) DIBAL-H, CH_2_Cl_2_, −78°C, 3****h; d) NaBH(OAc)_3_, CH_2_Cl_2_, rt, 8****h; e) 6NHCl, EtOAc, rt, 3****h; f) EDC.HCl, CH_2_Cl_2_, rt, 6****h.

**Table 1:** Effects of different substituents R^1^–R^4^ and link between ring 2 and 3 on CCR5 fusion activity.

Compd	R^1^	R^2^	R^3^	R^4^	inhibition rate (10 µM) a,b	IC_50_ (µM) a,b
**9a**	H	H	H	CH_3_	47%	–
**9b**	H	H	F	CH_3_	35%	–
**9c**	H	H	F	F	41%	–
**9d**	H	H	H	F	26%	–
**9e**	H	H	F	CN		0.64
**9f**	Cl	H	F	CN		3.70
**9g**	Cl	Cl	F	CN		10.01
**9h**	H	H	F	CONH_2_	20%	–
**13a**					inactive	–
**13b**					22%	–
**13c**					inactive	–
**13d**					25%	–
**17**					inactive	–

a Mean value of at least two experiments.

b DMSO as a negative control.

Based on the ‘Y shape’ pharmacophore model, a series of 1,3-diamine compounds that combined the structural features of maraviroc and TAK-220 (**1** and **2**) were designed by using a fragment- assembly strategy and bioisosteric-replacement principle. Hydrophobic aromatic rings with a variety of other functional groups were introduced to study structure-activity relationship of target compounds. Although some piperazine-based compounds have been reported before, the structures of compounds involved here are different from that of the reported papers [8]–[10]. Herein, we report the design, synthesis, and biological evaluation of the novel piperazine derivatives **9a–h, 13a–d**, **17**, and **23a–h**, with a goal to discover novel compounds as potential CCR5 antagonists for HIV treatment.

Synthesis of **13a–d** and **17** is depicted in Figure **5**. Reaction of piperazine with 2-(4-chlorophenyl)acetonitrile afforded *N*-arylation compound **11a**, which was submitted to reductive amination with aldehyde **7a** in the presence of sodium triacetoxyborohydride to provide **13a**. Compounds **11b–c** were achieved by treatment with 4-cyanobenzoyl chloride/4-(chloromethyl)benzonitrile and piperazine, respectively. Compounds **13b–c** were prepared from the corresponding intermediates **11b–c** following the same procedure as described for the preparation of **13a** from **11a**. Target compound **13d** was obtained by condensation mono-substituted piperazine **8c** with β**-**amino acid **12**, which was prepared by the acylation of β**-**phenylalanine **4a** with 4-fluorobenzoyl chloride. Target compound **17** was obtained as follows. Reaction of aniline **14** with 1-bromo-3-chloropropane under microwave irradiation afforded compound **15**. Acylation of **15** with 4-fluorobenzoyl chloride gave amide **16**, whose reaction with 4-substituted piperazine **8c** afforded target compound **17**.

Target compounds **23a–h** were synthesized through procedures as illustrated in Figure **6**. Compound **18** was prepared by esterification of **4a**, and then protected by BOC group to get ester **19**, which was reduced to the required aldehyde **20** using DIBAL-H, and then reductive amination with the compound **8c** furnished the key BOC-protected intermediate **21**. A BOC-deprotection step gave precursor **22**, whose acylation with corresponding carboxylic acids afforded target compounds **23a–h**.

### Biological Evaluation

A total of 21 novel piperazine derivatives **9a–h**, **13a–d**, **17**, and **23a–h** were screened for their inhibitory activity against cell-cell fusion between target cells expressing CD4/CCR5 and effector cells expressing the envelope protein of HIV-1, gp-120 (fusion assay) [13]. All synthesized analogs, which were firstly tested in MTT assay, showed no significant cytotoxicity against HEK 293 cells at a concentration of 50 µM.

Among the first set of compounds with three hydrophobic phenyl rings and varied substituents on phenyl rings (Table 1), compound **9e** exhibited the most potent CCR5 fusion activity with an IC_50_ value of 0.64 µM. The data in Table **1** clearly showed limited tolerance toward different substitutions (R_4_) at *p*-position of hydrophobic aromatic ring, as only compounds with a cyano-substituent retained good activity (**9e–9g**, IC_50_∶0.64 to 10.01 µM). Replacement of the *p*-cyano group with methyl (**9b**), fluoro (**9c**), or amide (**9h**) resulted in a sharp loss of potency. Replacement of the 4-fluorophenyl ring (hydrophobic aromatic ring 1) of **9b** and **9c** with phenyl group (**9a** and **9d**) did not significantly improve *in vitro* fusion potency. These results indicated that the presence of a cyano substituent at R^4^ position is favorable for CCR5 fusion activity.

Thus, a further optimization process was initiated with the introduction of a chlorine atom at R^1^ position or two chlorine atoms both at R^1^ and R^2^ positions of the phenyl ring 2 of **9e**. Compound **9f** with monochloro-substitution at R^1^ position showed 5-fold less potent activity than that of **9e**. 3,4-Dichloro-substituted analog (**9g**) demonstrated more than 15-fold less potent fusion activity in comparison to **9e**. These results suggested that introduction of a more hydrophobic function to the R^1^ and R^2^ positions is not favorable for fusion potency.

We then turned our attention to the link between ring 2 and ring 3 of compound **9e**, modifying the two basic nitrogen-atoms in piperazine and 4-cyanophenyl group. Replacing the cyano group at R^4^ with an acetonitrile group provided compound **13a**, which was found inactive on CCR5-mediated fusion assay at a concentration of 10 µM. Subsequent replacement of the 4-cyanophenyl group with 4-cyanobenzyl group afforded compound **13c**, which was also inactive in fusion assay. A carbonyl group was introduced in the right (**13b**) or left (**13d**) side of the piperazine to reduce the basicity of the piperazine. Neither **13b** nor **13d** showed potent CCR5 fusion activity. Our observation suggested that 4-(piperazin-1-yl)benzonitrile is a favored scaffold for CCR5 antagonists. Interestingly, replacement of the phenyl ring 2 from the carbon atom to the nitrogen atom (**17**) resulted in a substantial loss in CCR5 fusion activity.

Modifications as shown in Table **2** were also made to explore SARs of target compounds. Replacement of the fluoro-substitution at phenyl ring in ring 1 with a hydrogen (**23a**, IC_50_ = 2.44 µM) or a chlorine atom (**23b**, IC_50_ = 2.87 µM) resulted in a 4- or 5-fold reduction in CCR5-mediated fusion activity. Conversion of the fluoro-substituent of **9e** to a trifluoromethyl group (**23c**) led to a 15-fold loss in potency (IC_50_ = 10.00 µM). *p*-Isopropyl replacement (**23d**) was found to lead an even more sharp decrease in potency. These observations indicated that the electron-withdrawing group at the 4-position in ring 1 rather than the corresponding electron-donating group contribute significantly in fusion activity. Not as expected, introducing of N-acetyl-piperidin group, effective in compound **2**, to R^5^ position (**23e**) resulted in a complete loss in fusion activity. So did benzoylpiperdine derivatives (**23f**). Compound **23g** with a five-membered furan ring was found to be inactive in CCR5-mediated fusion assay. It was interesting that compound **23h** containing an aliphatic six-membered ring instead of the aromatic ring was found to be well-tolerated (IC_50_ = 6.29 µM). As such, hydrogen- or halogen-substituted phenyl ring and cyclohexyl group were identified as the optimal substituents.

**Table 2:** Modification of the *p*-fluorophenyl moiety of **9e**.

Compd	R^5^	inhibition rate (10 µM)a,b	IC_50_ (µM)a,b
**23a**	phenyl-		2.44
**23b**	4-chlorophenyl-		2.87
**23c**	4-trifluoromethylphenyl-		10.00
**23d**	4-isopropylphenyl-	20%	–
**23e**	1-acetylpiperidin-4-yl -	inactive	–
**23f**	1-benzoylpiperidin-4-yl-	15%	–
**23g**	furan-2-yl	inactive	–
**23h**	cyclohexyl-		6.29

a Mean value of at least two experiments.

b DMSO as a negative control.

Fluorophenyl analogs **9e**–**g**, phenyl, chlorophenyl, and trifluorophenyl analog **23a**–**c**, and cyclohexyl analog **23 h** were evaluated for their anti-HIV activity using a recombinant HIV-1 virus pseudotyped with the envelope proteins of the CCR5-tropic virus (HIV-1 single cycle antiviral assay) [14]. For overall estimation of the therapeutic potential of these novel CCR5 antagonists, maraviroc was used as a positive control. Results are shown in Tables **3**. Albeit the observation of potent activity in fusion assay, compound **9e** showed weak anti-HIV activity at a concentration of 10 µM. Compound **23 h** showed potent anti- HIV activity in comparison with the other tested piperazine derivatives.

**Table 3:** Antiviral activity of **9e**–**g**, **23a**–**c**, and **23h**.

Compd	inhibition rate (10 µM) a,b	IC_50_ (µM) a,b
**9e**	23.8%	–
**9f**	inactive	–
**9g**	2.9%	–
**23a**	28.9%	–
**23b**	29.9%	–
**23c**	24.6%	–
**23h**	66.5%	0.44
**Maraviroc**	96.8%	0.0011

a Mean value of two experiments.

b DMSO as a negative control.

### Conclusions

A novel series of piperazine derivatives were synthesized by adopting a fragment-assembly strategy and the bioisosteric-replacement principle. Target compounds were evaluated for their CCR5-mediated fusion activity and cytotoxicity, which showed that five compounds displayed medium to potent CCR5 antagosnist activity. Compound **23 h** was evaluated as a CC5 antagonist with an IC_50_ value of 6.29 µM and an antiviral agent with an IC_50_ value of 0.44 µM. The piperazine derivatives developed in this study, as well as the concluded SAR, might be useful for following optimization toward the development of novel CCR5 antagonists for HIV treatment.

## Materials and Methods

### Cell Cytotoxicity Assays

Cytotoxicities of target compounds were tested by using MTT (Sigma-Aldrich) assay. Briefly, 100 µl of HEK293 cell suspension (5000 cells/well) were dispensed in a 96-well plate, and then pre-incubated the plate for 24 hours at 37°C in 5% CO_2_. 10 µl of various concentrations of substances to be tested were added to the plate. After 7 hours incubation, 10 µl of CCK-8 solution were added to each well of the plate. The plate was incubated at 37°C for another 2 hours in the incubator, the optical absorbance was measured at 430 nm using a microplate reader.

### Two Stable Cell Lines

Effector cell line: This cell line express HIV envelope protein gp160 and chimera protein Rn-Dn. Rn-Dn is consist of the N terminal of renilla luciferase and the N terminal of DnaE intein from Anacystis nidulans R2 PCC7942.

Target cell line: This cell line express chemokine receptor 5(CCR5), CD4 protein and chimera protein Dc-Rc. Dc-Rc is consist of the C teminal of renilla luceferase and the C teminal of NnaE intein from Anacystis nidulans R2 PCC7942.

### Cell-cell Fusion Assay (CCF Assay)

The cell-cell fusion assay was performed as described previously [13].The effector cells were plated in 24 well white culture plates at 7.5×10^4^ cell per well in DMEM supplemented with 10% FBS, 800 µg/mL G418. The target cells in the growth medium were then added to the plates at 7.5×10^4^ cells/50 µL/well and incubated for 5 hours. At the end of coculture, 70 µL of renilla luceferase assay lysis was added into each well, and the cultures were gently shaken for 15 min. At the same time, add 20 µl of Renilla Luciferase Assay Reagent to the luminometer tube. Add 20 µl of cell lysate to the tube. Mix quickly by flicking the tube for 1 second. Place the tube in FB12 luminometer and initiate measurement. Luminescence was integrated over 1 second with a 2-second delay. When small molecule compounds needed to be added to the CCF assay system, the compounds were diluted manually in DMSO. Then, 10 µL of the diluted compounds was added to the effector cells just before the addition of target cells, thus making the final concentration of DMSO in the coculture 0.5%.

### Plasmids

HIV-1 proviral indicator construct pNL-Luc-E- contains a full-length HIV-1 genome, in which env was replaced by firefly Luciferase coding sequence. pENV-Ad8 expresses R5-tropic envelope (AD8).

### Viral Infectivity Assays

Single-cycle HIV-1 replication assays were performed as described previously [14]. In brief, 4×10^5^ 293T cells were co-transfected with 0.4 µg of pNL-Luc-E- and 0.4 µg of pENV-R5. After 48****h, the supernatant containing pseudovirion was harvested by filtration through a 0.45 µm filter and the amount of viral capsid protein was measured by p24 antigen capture ELISA (Biomerieux). The resultant supernatant (10 µL) was used to infect SupT1 cells (1×10^5^) in 96-well plates in the presence of testing compound at the concentration indicated. The SupT1 cells were lysed 48****h post-infection and firefly luciferase activities were determined using a firefly Luciferase Assay System (Promega). Values were normalized to the control group treated with DMSO, and represented relative infectivity of each sample testing.

### Supporting Information

Experimental protocols, NMR data (^1^H and ^13^C), Mass spectrometry data (MS and HRMS) and Melting points data of compounds are presented in File S1.

## Supporting Information

File S1 **Experimental protocols, NMR data (1H and 13C), Mass spectrometry data (MS and HRMS) and Melting points data of compounds.**
(DOC)

## References

[1] Yerly S, Kaiser L, Race E, Bru JP, Clavel F, et al (1999) Transmission of antiretroviral-drug- resistant HIV-1 variants. Lancet 354: 729–733. 10475184 10.1016/S0140-6736(98)12262-6

[2] Combadiere C, Ahuja SK, Tiffany HL, Murphy PM (1996) Cloning and functional expression of CC CKR5, a human monocyte CC chemokine receptor selective for MIP-1β, MIP-1α, and RANTES. J. Leukocyte Biol 60: 147–152. 8699119 10.1002/jlb.60.1.147

[3] Deng H, Liu R, Ellmeier W, Choe S, Unutmaz D, et al (1996) Identification of a major co-receptor for primary isolates of HIV-1. Nature 381: 661–666. 8649511 10.1038/381661a0

[4] Rodger DM, Richard MN (2008) Reviews of antiinfective agents: maraviroc: the first of a new class of antiretroviral agents. Clin Infect Dis 47: 236–241. 18532888 10.1086/589289

[5] Takashima K, Miyake H, Kanzaki N, Tagawa Y, Wang X, et al (2005) Highly potent inhibition of human immunodeficiency virus type 1 replication by TAK-220, an orally bioavailable small-molecule CCR5 antagonist. Antimicrob Agents Ch 49: 3474–3482. 10.1128/AAC.49.8.3474-3482.2005 PMC1196284 16048963

[6] Nishikawa M, Takashima K, Nishi T, Furuta RA, Kanzaki N, et al (2005) Analysis of binding sites for the new small-molecule CCR5 antagonist TAK-220 on human CCR5. Antimicrob Agents Ch 49: 4708–4715. 10.1128/AAC.49.11.4708-4715.2005 PMC1280122 16251315

[7] Kondru R, Zhang J, Ji CH, Mirzadegan T, Rotstein D, et al (2008) Molecular interactions of CCR5 with major classes of small-molecule anti-HIV CCR5 antagonists. Mol Pharmacol 73: 789–800. 18096812 10.1124/mol.107.042101

[8] Dong MX, Lu L, Li H, Wang X, Lu H, et al. (2012) Design, synthesis, and biological activity of novel 1,4-disubstituted piperidine/piperazine derivatives as CCR5 antagonist-based HIV-1 entry inhibitors. Bioorg Med Chem Lett 22; 3284–3286. 10.1016/j.bmcl.2012.03.019 22464131

[9] Feng DZ, Song YL, Jiang XH, Chen L, Long YQ (2007) Forward- and reverse-synthesis of piperazinopiperidine amide analogs: a general access to structurally diverse 4-piperazinopiperidine-based CCR5 antagonists. Org Biomol Chem 5: 2690–2697. 18019544 10.1039/b707175b

[10] Jiang XH, Song YL, Long YQ (2004) Facile synthesis of 4-substituted-4-aminopiperidine derivatives, the key building block of piperazine-based CCR5 antagonists. Bioorg Med Chem Lett 14: 3675–3678. 15203141 10.1016/j.bmcl.2004.05.014

[11] Torre O, Gotor-Fernández V, Gotor V (2006) Lipase-catalyzed resolution of chiral 1,3-amino alcohols: application in the asymmetric synthesis of (S)-dapoxetine. Tetrahedron: Asymmetry 17: 860–866.

[12] Weng ZY, Gao YP, Zhang JK, Dong XW, Liu T (2011) Synthesis and biological evaluation of novel N-[3-(4-phenylpiperazin-1-yl)-propyl]-carboxamide derivatives. J Chem Res 43–46.

[13] Chen LJ, Zhang YP, Li G, Huang HS, Zhou NM (2010) Functional characterization of a naturally occurring trans-splicing intein from Synechococcus elongatus in a mammalian cell system. Anal Biochem 407: 180–187. 20727340 10.1016/j.ab.2010.08.018

[14] Coakley E, Petropoulos C, Whitcomb J (2005) Assessing chemokine co-receptor usage in HIV. Curr Opin Infect Dis 18: 9–15. 15647694 10.1097/00001432-200502000-00003

