# Supplementary material for: Design, Synthesis and Biological Evaluation of Novel Piperazine Derivatives as CCR5 Antagonists
Source: PLoS One. 2013 Jan 7;8(1):e53636. doi: 10.1371/journal.pone.0053636 (PMC3538727; doi:10.1371/journal.pone.0053636)
Supplement: File S1 — Experimental protocols, NMR data (1H and 13C), Mass spectrometry data (MS and HRMS) and Melting points data of compounds. (DOC) [file pone.0053636.s001.doc]

**Supporting information**

Experimental protocols, NMR data (^1^H and ^13^C), Mass spectrometry data (MS and HRMS) and Melting points data of compounds are reported below.

Melting points were obtained on a Buchi B-540 apparatus (Buchi Labortechnik, Flawil, Switzerland) and are uncorrected. All ^1^H-NMR spectra were recorded on Bruker 500 MHz-spectrometer (Bruker Bioscience, Billerica, MA, USA) with SiMe_4_ as the internal standard in CDCl_3_ and D_2_O. All ^13^C-NMR spectra were recorded on a Brüker AM 400 instrument. Chemical shifts were reported in* δ* values (ppm), relative to internal TMS, and *J* values were reported in Hertz (Hz). Mass spectra (ESI, positive ion) were recorded on an Esquire-LC-00075 spectrometer (Bruker Bioscience). HRMS were performed on an Agilent 6224 TOF LC/MS instrument. Reagents and solvents were of commercial quality, which were purchased from known commercial suppliers and were used without further purification.

**General procedure for the preparation of compounds 4a-c.**

A similar procedure to the one described by Oliver Torre *et al* was followed with some modifications. The corresponding benzaldehyde **3a-c** (30.0 mmol), ammonium acetate (3.1 g, 40.2 mmol) and malonic acid (3.1 g, 30.2 mmol) were refluxed in EtOH (25 mL) for 12 h. The reaction mixture was cooled to room temperature and the white solid was collected by filtration washing with Et_2_O (30 mL). The solid was recrystallized in a mixture MeOH/H_2_O (10:1) affording **4a-c** as a crystalline solid.

**3-Amino-3-phenylpropanoic acid (4a)**. White solid (72%), m.p.: 218-220 ^o^C. ^1^H-NMR (500 MHz, D_2_O): *δ*: 7.46-7.38(m, 5H, Ar-H), 4.34-4.31(m, 1H, CH), 2.71-2.58(m, 2H, CH_2_). ESI-MS *m/z*: 166 [M+H]^+^.

**3-Amino-3-(4-chlorophenyl)propanoic acid (4b).** White solid (68%), m.p.: 242-244 ^o^C. ^1^H-NMR (500 MHz, D_2_O): *δ*: 7.49-7.41(m, 4H, Ar-H), 4.35-4.32(m, 1H, CH), 2.71-2.59(m, 2H, CH_2_). ESI-MS *m/z*: 200 [M+H]^+^.

**3-Amino-3-(3,4-dichlorophenyl)propanoic acid (4c).** White solid (65%), m.p.: 249-255 ^o^C. ^1^H-NMR (500 MHz, D_2_O): *δ*: 7.51-7.46(m, 3H, Ar-H), 4.34-4.32(m, 1H, CH), 2.74-2.60(m, 2H, CH_2_). ESI-MS *m/z*: 234 [M+H]^+^.

**General procedure for the preparation of compounds 5a-c.**

A similar procedure to the one described by Oliver Torre *et al* was followed with some modifications. A solution of **4a-c** (5.6 mmol) in dry THF (20 mL) was cooled to 0 ^o^C and LiAlH_4_ (636 mg, 16.8 mmol) was added in small portions. The reaction mixture was refluxed for 2 h following the disappearance of the starting material by TLC analysis. The reaction mixture was then cooled to 0 ^o^C, and the excess hydride destroyed by adding H_2_O dropwise. The grey mixture was extracted in EtOAc (3×20 mL) and the organic phases were combined, washed with brine (2×10 mL), dried (Na_2_SO_4_) and evaporated under reduced pressure to get compounds **5a-c**.

**3-Amino-3-phenylpropan-1-ol (5a).** White solid (75%), m.p.: 75-77 ^o^C. ^1^H-NMR (500 MHz, CDCl_3_): *δ*: 7.38-7.24(m, 5H, Ar-H), 4.24-4.19(m, 1H, CH), 3.71-3.55(m, 2H, CH_2_), 1.92-1.86(m, 2H, CH_2_). ESI-MS *m/z*: 152 [M+H]^+^.

**3-Amino-3-(4-chlorophenyl)propan-1-ol (5b).** White solid (65%), m.p.: 54-56 ^o^C. ^1^H-NMR (500 MHz, CDCl_3_): *δ*: 7.48-7.39(m, 4H, Ar-H), 4.25-4.19(m, 1H, CH), 3.71-3.59(m, 2H, CH_2_), 1.94-1.87(m, 2H, CH_2_). ESI-MS *m/z*: 186 [M+H]^+^.

**3-Amino-3-(3,4-dichlorophenyl)propan-1-ol (5c).** Yellow oil (78%). ^1^H-NMR (500 MHz, CDCl_3_): *δ*: 7.51-7.42(m, 3H, Ar-H), 4.26-4.19(m, 1H, CH), 3.73-3.60(m, 2H, CH_2_), 1.90-1.85(m, 2H, CH_2_). ESI-MS *m/z*: 220 [M+H]^+^.

**General procedure for the preparation of compounds 6a-d.**

To an ice-cooled stirred suspension of compounds **5a-c** (3 mmol) in CH_2_Cl_2_ (3 mL), was added Et_3_N (1.2 mL, 9 mmol) followed by the corresponding acyl chloride (3.6 mmol). After being stirred at 0 ^o^C for 4 h, the mixture was diluted with saturated aqueous NaHCO_3_ (30 mL), and the organic layer was separated. The aqueous layer was extracted with CH_2_Cl_2_ (30 mL), and the combined organic layer was washed with brine (2×20 mL), dried (Na_2_SO_4_), filtered, and concentrated in vacuo. The residue was purified by column chromatography (petroleum ether : ethyl acetate = 1 : 1) to afford the product **6a-d**.

**4-Fluoro-*N*-(3-hydroxy-1-phenylpropyl)-benzamide (6a).** White solid (88%). m.p.: 90-92 ^o^C. ^1^H-NMR (500 MHz, CDCl_3_): *δ*: 7.97-7.25(m, 9H, Ar-H), 7.05(d, 1H, *J* = 7.0 Hz, NH), 5.40-5.36(m, 1H), 3.72-3.65(m, 2H), 3.65(s, 1H, OH), 2.24-2.19(m, 1H), 1.95-1.90(m, 1H). ESI-MS *m/z*: 274 [M+H]^+^.

***N*-(1-(4-Chlorophenyl)-3-hydroxypropyl)-4-fluorobenzamide (6b).** Pale yellow solid (83%). m.p.: 113-115 ^o^C. ^1^H-NMR (500 MHz, CDCl_3_): *δ*: 7.93-7.28(m, 8H, Ar-H), 7.05(d, 1H, *J* = 7.0 Hz, NH), 5.40-5.36(m, 1H), 3.77-3.73(m, 2H), 3.64(s, 1H, OH), 2.24 -2.20(m, 1H), 1.93-1.90(m, 1H). ESI-MS *m/z*: 308 [M+H]^+^.

***N*-(1-(3,4-Dichlorophenyl)-3-hydroxypropyl)-4-fluorobenzamide (6c).** Pale yellow solid (90%). m.p.: 98-102 ^o^C. ^1^H-NMR (500 MHz, CDCl_3_): *δ*: 7.85-7.25(m, 7H, Ar-H), 7.03(d, 1H, *J* = 7.0 Hz, NH), 5.44-5.40(m, 1H), 3.77-3.74(m, 2H), 3.69(s, 1H, OH), 2.24-2.21(m, 1H), 1.95-1.92(m, 1H). ESI-MS *m/z*: 342 [M+H]^+^.

***N*-(3-Hydroxy-1-phenylpropyl)-benzamide (6d).** Pale yellow solid (90%), R_f_ = 0.50, m.p.: 85-87 ^o^C. ^1^H-NMR (500 MHz, CDCl_3_): *δ*: 7.77-7.26(m, 10H, Ar-H), 7.11(d, 1H, *J* = 7.0 Hz, NH), 5.42-5.38(m, 1H), 3.75-3.69(m, 2H), 3.42(s, 1H, OH), 2.22-2.18(m, 1H), 1.97-1.92(m, 1H). ESI-MS *m/z*: 256 [M+H]^+^.

**General procedure for the preparation of compounds 7a-d.**

To a solution of oxalyl chloride (0.26 mL, 3.0 mmol) in 20 mL CH_2_Cl_2_ at -78 ^o^C was added a solution of dimethyl sulfoxide (0.5 mL, 6.0 mmol) in 8 mL CH_2_Cl_2_. After addition was complete, the mixture was stirred for 3 min before the addition of a solution of compounds **6a-d** (2.8 mmol) in 8 mL CH_2_Cl_2_. After 40 min, triethylamine (2.0 mL, 14.0 mmol) was added. The mixture was stirred for 10 min and then allowed to warm to room temperature. After 1 h, the reaction mixture was partitioned between H_2_O (10 mL) and CH_2_Cl_2_ (20 mL). The combined organic layer was washed with brine (2×10 mL), dried (Na_2_SO_4_), filtered, and concentrated in vacuo. The residue was purified by column chromatography (petroleum ether : ethyl acetate = 2 : 1) to afford the product **7a-d**.

**4-Fluoro-*N*-(3-oxo-1-phenylpropyl)-benzamide (7a).** Pale yellow solid (90%). m.p.: 85-88 ^o^C. ^1^H-NMR (500 MHz, CDCl_3_): *δ*: 9.53(s, 1H, CHO), 7.95-7.26(m, 9H, Ar-H), 6.82(d, 1H, *J* = 7.0 Hz, NH), 5.68-5.64(m, 1H), 3.25-3.21(m, 1H), 3.09-3.06(m, 1H). ESI-MS *m/z*: 272 [M+H]^+^.

***N*-(1-(4-Chlorophenyl)-3-oxopropyl)-4-fluorobenzamide (7b).** Yellow solid (92%). m.p.: 127-129 ^o^C. ^1^H-NMR (500 MHz, CDCl_3_): *δ*: 9.56(s, 1H, CHO), 7.91-7.30(m, 8H, Ar-H), 6.95(d, 1H, *J* = 7.0 Hz, NH), 5.73-5.67(m, 1H), 3.21-3.17(m, 1H), 3.12-3.07(m, 1H). ESI-MS *m/z*: 306 [M+H]^+^.

***N*-(1-(3,4-Dichlorophenyl)-3-oxopropyl)-4-fluorobenzamide (7c).** Yellow solid (85%). m.p.: 106-110 ^o^C. ^1^H-NMR (500 MHz, CDCl_3_): *δ*: 9.59(s, 1H, CHO), 7.81-7.35(m, 7H, Ar-H), 6.98(d, 1H, *J* = 7.0 Hz, NH), 5.75-5.69(m, 1H), 3.21-3.18(m, 1H), 3.19-3.14(m, 1H). ESI-MS *m/z*: 340 [M+H]^+^.

***N*-(3-Oxo-1-phenylpropyl)-benzamide (7d).** Pale yellow solid (92%). m.p.: 112-114 ^o^C. ^1^H-NMR (500 MHz, CDCl_3_): *δ*: 9.74(s, 1H, CHO), 7.76-7.30(m, 10H, Ar-H), 6.89(d, 1H, *J* = 7.0 Hz, NH), 5.73-5.69(m, 2H), 3.24-3.19(m, 1H), 3.09-3.05(m, 1H). ESI-MS *m/z*: 254 [M+H]^+^.

**General method for synthesis of compounds (9a-h).**

A mixture of compound **7** (1 mmol), compound **8** (1 mmol), and Et_3_N (0.10g, 1 mmol) in anhydrous CH_2_Cl_2_ (10 mL) was stirred for 0.5 h at room temperature. Then NaBH(OAc)_3_ (0.64g, 3 mmol) was added, and the mixture was continued to stir for 8 h. The reaction was quenched by the addition of saturated NaHCO_3_ (10 mL) and the solution was extracted with CH_2_Cl_2_ (3×10 mL). The organic layer was washed with brine (2×10 mL), dried (Na_2_SO_4_), filtered, and concentrated in vacuo. The residue was purified by column chromatography (petroleum ether : ethyl acetate = 1 : 2) to afford the product **9a-h**.

***N*-(1-Phenyl-3-(4-*p*-tolylpiperazin-1-yl)propyl)benzamide (9a).** Yellow solid (60%). m.p.: 165-167 ^o^C. ^1^H-NMR (500 MHz, CDCl_3_): *δ*: 8.91(d, 1H, *J* = 6.0 Hz, NH), 7.91-6.84(m, 14H, Ar), 5.38-5.35(m, 1H), 3.23-3.16(m, 4H, piperazinyl-H), 2.74-2.60(m, 4H, piperazinyl-H), 2.60-2.57(m, 1H), 2.47-2.44(m, 1H), 2.31(s, 3H, CH_3_), 2.28-2.23(m, 2H). ^13^C-NMR (100 MHz, CDCl_3_): *δ*: 166.81, 149.04, 142.20, 134.62, 131.45, 129.72, 128.60, 128.56, 128.47, 127.23, 127.02, 126.17, 116.64, 55.01, 53.87, 53.48, 49.84, 31.41, 20.48. ESI-MS *m/z*: 414 [M+H]^+^. HRMS [M+H]^+^ Calcd for C_27_H_31_N_3_O: 414.2467, found: 414.2459.

**4-Fluoro-*N*-(1-phenyl-3-(4-*p*-tolylpiperazin-1-yl)propyl)benzamide (9b).** Yellow solid (62%). m.p.: 171-173 ^o^C. ^1^H-NMR (500 MHz, CDCl_3_): *δ*: 8.85 (d, 1H, *J* = 6.0 Hz, NH), 7.95-6.90(m, 13H, Ar-H), 5.37-5.34(m, 1H), 3.21-3.13(m, 4H, piperazinyl-H), 2.72-2.59(m, 4H, piperazinyl-H), 2.61-2.57(m, 1H), 2.48-2.44(m, 1H), 2.30(s, 3H, CH_3_), 2.27-2.22(m, 2H). ^13^C-NMR (100 MHz, CDCl_3_): *δ*: 165.97, 165.73, 163.48, 148.97, 142.08, 130.85, 130.83, 129.83, 129.77, 129.53, 129.44, 128.58, 127.06, 126.13, 116.61, 115.56, 115.35, 55.06, 54.07, 53.52, 49.97, 31.34, 20.45. ESI-MS *m/z*: 432 [M+H]^+^. HRMS [M+H]^+^ Calcd for C_27_H_30_FN_3_O: 432.2373, found: 432.2365.

**4-Fluoro-*N*-(3-(4-(4-fluorophenyl)piperazin-1-yl)-1-phenylpropyl)benzamide (9c).** Yellow solid (53%). m.p.: 157-159 ^o^C. ^1^H-NMR (500 MHz, CDCl_3_): *δ*: 8.74(d, 1H, *J* = 6.0 Hz, NH), 7.95-6.85(m, 13H, Ar-H), 5.36-5.32(m, 1H), 3.24-3.15(m, 4H, piperazinyl-H), 2.82-2.68(m, 4H, piperazinyl-H), 2.67-2.59(m, 2H), 2.28-2.25(m, 1H), 2.11-2.06(m, 1H). ^13^C-NMR (100 MHz, CDCl_3_): *δ*: 166.01, 165.84, 163.50, 158.78, 156.40, 147.50, 141.90, 130.73, 130.70, 129.61, 129.52, 128.67, 127.24, 126.19, 118.23, 118.16, 115.86, 115.64, 115.58, 115.37, 54.94, 53.73, 53.29, 50.12, 31.24. ESI-MS *m/z*: 436 [M+H]^+^. HRMS [M+H]^+^ Calcd for C_26_H_27_F_2_N_3_O: 436.2122, found: 436.2130.

***N*-(3-(4-(4-Fluorophenyl)piperazin-1-yl)-1-phenylpropyl)benzamide (9d).** Yellow solid (54%). m.p.: 152-154 ^o^C. ^1^H-NMR (500 MHz, CDCl_3_): *δ*: 8.75(d, 1H, *J* = 7.0 Hz, NH), 7.93-6.88(m, 14H, Ar-H), 5.40-5.37(m, 1H), 3.23-3.11(m, 4H, piperazinyl-H), 2.79-2.67(m, 4H, piperazinyl-H), 2.66-2.60(m, 1H), 2.55-2.50 (m, 1H), 2.31-2.26(m, 1H), 2.05-2.00(m, 1H). ^13^C-NMR (100 MHz, CDCl_3_): *δ*: 166.76, 158.87, 156.42, 147.63,141.91, 134.73, 131.55, 128.70, 128.49, 127.28, 127.13, 126.25, 118.31, 118.24, 115.60, 115.39, 54.84, 53.65, 53.29, 50.17, 31.42. ESI-MS *m/z*: 418 [M+H]^+^. HRMS [M+H]^+^ Calcd for C_26_H_28_FN_3_O: 418.2216, found: 418.2205.

***N*-(3-(4-(4-Cyanophenyl)piperazin-1-yl)-1-phenylpropyl)-4-fluorobenzamide (9e).** Yellow solid (67%). m.p.: 160-162 ^o^C. ^1^H-NMR (500 MHz, CDCl_3_): *δ*: 8.26(d, 1H, *J*=6.5Hz, NH), 7.86-6.85(m, 13H, Ar-H), 5.33-5.30(m, 1H), 3.38-3.29(m, 4H, piperazinyl-H), 2.69-2.57(m, 4H, piperazinyl-H), 2.56-2.43(m, 2H), 2.30-2.19(m, 1H), 2.08-2.01(m, 1H). ^13^C-NMR (400 MHz, CDCl_3_): *δ*: 166.02, 165.77, 163.51, 153.11, 141.77, 133.60, 130.86, 130.83, 129.44, 129.35, 128.74, 127.36, 126.28, 119.82, 115.66, 115.44, 114.44, 101.09, 55.00, 53.58, 52.87, 47.26, 31.68. ESI-MS *m/z*: 443 [M+H]^+^. HRMS [M+H]^+^ Calcd for C_27_H_27_FN_4_O: 443.2169, found: 443.2149.

***N*-(1-(4-Chlorophenyl)-3-(4-(4-cyanophenyl)piperazin-1-yl)propyl)-4-fluorobenzamide (9f).** Yellow solid (45%). m.p.: 121-123 ^o^C. ^1^H-NMR (500 MHz, CDCl_3_): *δ*: 8.63(d, 1H, *J* = 6.5 Hz, NH), 7.81-6.79(m, 12H, Ar-H), 5.39-5.35(m, 1H), 3.26-3.11(m, 4H, piperazinyl-H), 2.70-2.60(m, 4H, piperazinyl-H), 2.57-2.53(m, 1H), 2.49-2.44(m, 1H), 2.30-2.27(m, 2H). ^13^C-NMR (100 MHz, CDCl_3_): *δ*: 166.10, 165.87, 163.60, 153.02, 140.45, 133.63, 133.09, 130.57, 130.53, 129.48, 129.39, 128.89, 127.63, 119.75, 115.71, 115.49, 114.52, 101.31, 54.98, 53.29, 52.86, 47.22, 31.39. ESI-MS *m/z*: 477 [M+H]^+^. HRMS [M+H]^+^ Calcd for C_27_H_26_ClFN_4_O: 477.1779, found: 477.1760.

***N*-(3-(4-(4-Cyanophenyl)piperazin-1-yl)-1-(3,4-dichlorophenyl)propyl)-4-fluorobenzamide (9g).** Yellow solid (52%). m.p.: 85-88 ^o^C. ^1^H-NMR (500 MHz, CDCl_3_): *δ*: 8.62(d, 1H, *J* = 6.0 Hz, NH), 7.87-6.86(m, 11H, Ar-H), 5.26-5.23(m, 1H), 3.40-3.29(m, 4H, piperazinyl-H), 2.68-2.60(m, 4H, piperazinyl-H), 2.54-2.40(m, 3H), 2.15-2.08(m, 1H). ^13^C-NMR (100 MHz, CDCl_3_): *δ*: 166.13, 165.59, 163.63, 153.29, 141.02, 133.55, 133.56, 130.91, 130.54, 130.51, 130.11, 129.57, 129.47, 128.99, 127.63, 119.77, 115.77, 115.54, 114.68, 101.21, 54.89, 53.22, 52.76, 47.20, 31.12. ESI-MS *m/z*: 511 [M+H]^+^. HRMS [M+H]^+^ Calcd for C_27_H_25_Cl_2_FN_4_O: 511.1389, found: 511.1395.

***N*-(3-(4-(4-Carbamoylphenyl)piperazin-1-yl)-1-phenylpropyl)-4-fluorobenzamide (9h).** Pale yellow solid (65%). m.p.: 185 ^o^C (dec). ^1^H-NMR (500 MHz, CDCl_3_): *δ*: 8.53(d, 1H, *J* = 6.0 Hz, NH), 7.87-6.87(m, 13H, Ar-H), 5.71(s, 2H, NH_2_), 5.36-5.33(m, 1H), 3.37-3.26(m, 4H, piperazinyl-H), 2.70-2.54(m, 4H, piperazinyl-H), 2.56-2.54(m, 1H), 2.46-2.42(m, 1H), 2.25-2.20(m, 2H). ^13^C-NMR (100 MHz, CDCl_3_): *δ*: 170.83, 170.65, 167.16, 165.95, 154.39, 142.29, 129.53, 129.44, 128.71,128. 128.13, 126.84, 126.20, 122.91, 115.00, 114.78, 113.72, 55.01, 52.69, 52.57, 47.37, 32.05. ESI-MS *m/z*: 461 [M+H]^+^. HRMS [M+H]^+^ Calcd for C_27_H_29_FN_4_O_2_: 461.2275, found: 461.2290.

**Synthesis of 2-(4-(piperazin-1-yl)phenyl)acetonitrile (11a).**

A solution of 2-(4-chlorophenyl)acetonitrile (0.15g, 1 mmol) in DMSO (1 mL) was added to a refluxing solution of piperazine (0.86g, 10 mmol) and K_2_CO_3_ (0.27g, 2 mmol) in DMSO (3 mL) over 2 minutes. The mixture was maintained at reflux for 18 h, then cooled to room temperature and extracted with 2N hydrochloric acid (3×10 mL). The aqueous layer was basified with 10% aqueous NaOH solution and extracted with ethyl acetate (3×15 mL). The combined organic layer was washed with brine (2×10 mL), dried (Na_2_SO_4_), filtered, and concentrated in vacuo. The residue was purified by column chromatography (ethyl alcohol : ethyl acetate = 20 : 1) to afford the product **12a** as yellow oil (0.11g, 55%).

^1^H-NMR (500 MHz, CDCl_3_): *δ*: 7.50-7.13(m, 4H, Ar), 3.43(s, 2H, CH_2_), 3.27-3.20 (m, 4H, piperazinyl-H), 3.07-2.98(m, 4H, piperazinyl-H), 2.01(m, 1H, NH). ESI-MS *m/z*: 202 [M+H]^+^.

**Synthesis of 4-(piperazine-1-carbonyl)benzonitrile (11b).**

To a solution of piperazine (0.86g, 10 mmol) and Et_3_N (0.20g, 2 mmol) in CH_2_Cl_2_ (15 mL), a solution of 4-cyanobenzoyl chloride (0.33g, 2 mmol) in CH_2_Cl_2_ (10 mL) was added dropwise. The reaction mixture was stirred for about 3h at room temperature, and extracted with 2N hydrochloric acid (3×10 mL). The aqueous layer was basified with 10% aqueous NaOH solution and extracted with ethyl acetate (3×20 mL). The combined organic layer was washed with brine (2×10 mL), dried (Na_2_SO_4_), filtered, and concentrated in vacuo to get product **12b** as yellow oil (0.22g, 50%). ^1^H-NMR (500 MHz, CDCl_3_): *δ*: 7.55-7.18(m, 4H, Ar), 3.37-3.28 (m, 4H, piperazinyl-H), 3.19-3.05(m, 4H, piperazinyl-H), 1.98(m, 1H, NH). ESI-MS *m/z*: 216 [M+H]^+^.

**Synthesis of 4-(piperazin-1-ylmethyl)benzonitrile (11c).**

Piperazine (0.86g, 10 mmol) was added to THF (10 mL), and the mixture was heated to reflux until the piperazine was fully dissolved. Then the solution of 4-(chloromethyl)benzonitrile (0.30g, 2 mmol) in THF (3 mL) was added dropwise. The reaction mixture was refluxed for 2.5 h. The stirring mixture was cooled and then filtered. The solid was washed with THF (2×3 mL). The combined organic layer was concentrated in vacuo, which was then washed with 10% aqueous NaOH solution (2×5 mL). The aqueous layer was extracted with ethyl acetate (3×20 mL). The combined organic layer was washed with brine (2×10 mL), dried (Na_2_SO_4_), filtered, and concentrated in vacuo. The residue was purified by column chromatography (ethyl alcohol : ethyl acetate = 10 : 1) to afford the product **12c** as yellow oil (0.17g, 43%).

^1^H-NMR (500 MHz, CDCl_3_): *δ*: 7.58-7.20(m, 4H, Ar), 3.43(s, 2H, CH_2_), 3.17-3.08(m, 4H, piperazinyl-H), 3.00-2.89(m, 4H, piperazinyl-H), 1.88(m, 1H, NH). ESI-MS *m/z*: 202 [M+H]^+^.

The following compounds **13a-c** were prepared from compounds **11a-c** with **7a** by a method similar to that described for **9a-h**.

***N*-(3-(4-(4-(Cyanomethyl)phenyl)piperazin-1-yl)-1-phenylpropyl)-4-fluorobenzamide (13a).** Yellow solid (67%). m.p.: 115-119 ^o^C. ^1^H-NMR (500 MHz, CDCl_3_): *δ*: 8.09-7.05(m, 13H, Ar-H), 5.35-5.31(m, 1H), 3.93-3.60(m, 4H, piperazinyl-H), 3.41(s, 2H, CH_2_), 2.98-2.65(m, 4H, piperazinyl-H), 2.52-2.46(m, 2H), 2.24-2.16(m, 2H). ^13^C-NMR (125 MHz, CDCl_3_): *δ*: 165.79, 165.71, 163.72, 150.67, 142.00, 130.78, 130.75, 129.53, 129.46, 128.88, 128.64, 127.18, 126.16, 121.00, 118.29, 116.47, 115.56, 115.38, 54.96, 53.80, 53.18, 48.96, 31.45, 22.81. ESI-MS *m/z*: 457 [M+H]^+^. HRMS [M+H]^+^ Calcd for C_28_H_29_FN_4_O: 457.2325, found: 457.2301.

***N*-(3-(4-(4-Cyanobenzoyl)piperazin-1-yl)-1-phenylpropyl)-4-fluorobenzamide (13b)**. Yellow solid (68%). m.p.: 80-84 ^o^C. ^1^H-NMR (500 MHz, CDCl_3_): *δ*: 7.88-7.08(m, 13H, Ar-H), 5.34-5.30(m, 1H), 4.00-3.70(m, 4H, piperazinyl-H), 3.60-3.32(m, 4H, piperazinyl-H), 2.62-2.57(m, 2H), 2.20-2.13(m, 2H). ^13^C-NMR (100 MHz, CDCl_3_): *δ*: 173.34, 168.60, 168.19, 165.84, 141.48, 139.16, 132.62, 132.50, 129.37, 129.29, 128.84, 127.82, 127.78, 127.58, 126.35, 115.73, 115.52, 114.23, 113.87, 54.95, 53.09, 53.02, 52.87, 31.91. ESI-MS *m/z*: 471 [M+H]^+^. HRMS [M+H]^+^ Calcd for C_28_H_27_FN_4_O_2_: 471.2118, found: 471.2139.

***N*-(3-(4-(4-Cyanobenzyl)piperazin-1-yl)-1-phenylpropyl)-4-fluorobenzamide (13c).** Yellow solid (70%). m.p.: 83-87 ^o^C. ^1^H-NMR (500 MHz, CDCl_3_): *δ*: 9.07(d, 1H, *J* = 6.5 Hz, NH), 7.96-7.13(m, 13H, Ar-H), 5.36-5.32(m, 1H), 3.58(s, 2H, CH_2_), 2.73-2.42(m, 8H, piperazinyl-H), 2.45-2.39(m, 1H), 2.35-2.30(m, 2H), 2.02-1.98(m, 1H). ^13^C-NMR (100 MHz, CDCl_3_): *δ*: 166.07, 165.65, 163.57, 141.64, 141.19, 132.90, 130.59, 130.55, 129.38, 129.28, 128.91, 128.00, 126.67, 119.56, 115.75, 115.52, 129.54, 112.11, 62.19?, 54.58, 53.29, 52.64, 47.34, 31.99. ESI-MS *m/z*: 457 [M+H]^+^. HRMS [M+H]^+^ Calcd for C_28_H_29_FN_4_O: 457.2325, found: 457.2340.

**Synthesis of 3-(4-fluorobenzamido)-3-phenylpropanoic acid (12).**

To an ice-cooled stirred suspension of compound **4a** (0.50g, 3 mmol) in CH_2_Cl_2_ (10 mL), Et_3_N was added (1.2 mL, 9 mmol) followed by 4-fluorobenzoyl chloride (0.47g, 3.0 mmol). After being stirred at 0 ^o^C for 3 h, the mixture was extracted with 10% aqueous NaOH solution (3×20 mL). The aqueous layer was basified with 2N hydrochloric acid and extracted with ethyl acetate (3×20 mL). The combined organic layer was washed with brine (2×10 mL), dried (Na_2_SO_4_), filtered, and concentrated in vacuo to get product **12** as pale yellow solid (0.59g, 69%). m.p.: 165-168 ^o^C. ^1^H-NMR (500 MHz, CDCl_3_): *δ*: 8.89(d, 1H, *J* = 6.0 Hz, NH), 7.70-7.26(m, 9H, Ar-H), 5.45-5.43(m, 1H), 2.88-2.86(m, 1H), 2.78-2.75(m, 1H). ESI-MS *m/z*: 288 [M+H]^+^.

**Synthesis of *N*-(3-(4-(4-cyanophenyl)piperazin-1-yl)-3-oxo-1-phenylpropyl)-4-fluoro- benzamide (13d).**

A mixture of compound **12** (29mg, 0.1 mmol), compound **8c** (23mg, 0.1 mmol), Et_3_N (0.1 mmol), and 1-ethyl-3-(3-dimethylaminopropyl)-carbodiimide hydrochloride (EDC.HCl) (22 mg, 0.12 mmol) in CH_2_Cl_2_ (5 mL) was stirred at room temperature for 8 h. The mixture was evaporated in vacuo and purified by column chromatography on silica gel (petroleum ether : ethyl acetate = 1 : 2) to afford the product **13d** as yellow solid (42%). m.p.: 98-102 ^o^C. ^1^H-NMR (500 MHz, CDCl_3_): *δ*: 8.58(d, 1H, *J* = 6.5 Hz, NH), 7.65-6.89(m, 13H, Ar-H), 5.32-5.28(m, 1H), 3.95-3.40(m, 4H, piperazinyl-H), 3.21-3.02(m, 4H, piperazinyl-H), 2.49-2.37(m, 2H). ^13^C-NMR (100 MHz, CDCl_3_): *δ*: 171.37, 166.07, 165.28, 163.57, 153.06,141.49, 133.57, 130.52, 129.57, 129.48, 128.62, 127.41, 126.14, 119.75(CN), 115.64, 115.42, 114.58, 101.25, 50.54, 37.57, 37.50, 35.45. ESI-MS *m/z*: 457 [M+H]^+^. HRMS [M+H]^+^ Calcd for C_27_H_25_FN_4_O_2_: 457.1962, found: 457.1975.

**Synthesis of *N*-(3-Chloropropyl)-benzenamine (15)**

The aniline **14** (0.84g, 9 mmol), 1-bromo-3-chloropropane (0.49g, 3 mmol), and KI (0.05g, 0.3 mmol) in CH_3_CN (5mL) was kept under stirring at 110 ^o^C for 15 min using a Biotage microwave reactor. After cooling to room temperature, the mixture was filtered, and the filtrate was concentrated in vacuo. The residue was diluted with EtOAc (30mL), washed with water (30 mL) and brine (3×15 mL), dried (Na_2_SO_4_), filtered, and concentrated in vacuo. The residue was purified by column chromatography on silica gel (petroleum ether : ethyl acetate = 12 : 1) to afford the product **16** as yellow oil (0.34g, 67%). ^1^H-NMR (500 MHz, CDCl_3_): *δ*: 7.36-7.18(m, 2H, Ar-H), 7.16-6.99(m, 3H, Ar-H), 3.68-3.65(m, 2H, CH_2_), 3.60-3.56(m, 2H, CH_2_), 3.03-2.99(m, 2H, CH_2_). ESI-MS *m/z*: 170 [M+H]^+^.

**Synthesis of *N*-(3-chloropropyl)-4-fluoro-*N*-phenylbenzamide (16)**

To an ice-cooled stirred suspension of compound **15** (0.17g, 1 mmol) in CH_2_Cl_2_ (3 mL), was added Et_3_N (0.4mL, 3 mmol) followed by 4-fluorobenzoyl chloride (0.19g, 1.2 mmol), and the mixture was stirred at 0 ^o^C for 5 h. The mixture was diluted with saturated aqueous NaHCO_3_ (10 mL), and the organic layer was separated. The aqueous layer was extracted with CH_2_Cl_2_ (10 mL), and the combined organic layer was washed with brine (2×10 mL), dried (Na_2_SO_4_), filtered, and concentrated in vacuo. The residue was purified by column chromatography on silica gel (petroleum ether : ethyl acetate = 10 : 1) to afford the product **16** as white solid (0.26g, 90%). m.p.: 71-73 ^o^C. ^1^H-NMR (500 MHz, CDCl_3_): *δ*: 7.30-7.06(m, 5H, Ar-H), 7.01-6.99(m, 2H, Ar-H), 6.82-6.79(m, 2H, Ar-H), 4.05(t, *J* = 7.0 Hz, 2H, CH_2_), 3.60(t, *J* = 7.0 Hz, 2H, CH_2_), 2.18-2.13(m, 2H, CH_2_). ESI-MS *m/z*: 292 [M+H]^+^.

**Synthesis of *N*-(3-(4-(4-cyanophenyl)piperazin-1-yl)propyl)-4-fluoro-*N*-phenylbenzamide (17)**

A mixture of compound **16** (0.15g, 0.5 mmol), compound **8c** (0.5 mmol), KI (83 mg, 0.5 mmol), and K_2_CO_3_ (208 mg, 1.5 mmol) in MeCN (8 mL) was refluxed for 24 h. After cooling to room temperature, the mixture was concentrated in vacuo, the residue was diluted with water (5 mL), then extracted with EtOAc (3×10 mL). The organic layer was dried (Na_2_SO_4_), filtered, and concentrated in vacuo. The residue was purified by column chromatography on silica gel (petroleum ether : ethyl acetate = 1 : 1) to afford the product **17** as yellow solid (41%). m.p.: 82-84 ^o^C. ^1^H-NMR (500 MHz, CDCl_3_): *δ*: 7.48-7.02(m, 9H, Ar-H), 6.82-6.86(m, 4H, Ar-H), 3.98(m, 2H, CH_2_), 3.29(m, 4H, piperazinyl-H), 2.53(m, 4H, piperazinyl-H), 2.44(m, 2H, CH_2_), 1.78-1.74(m, 2H, CH_2_). ^13^C-NMR (100 MHz, CDCl_3_): *δ*: 166.11, 165.80, 163.60, 153.43, 140.87, 133.78, 130.77, 130.74, 129.49, 129.39, 128.55, 124.26, 120.18, 117.92, 115.71, 115.50, 114.69, 101.47, 54.55, 52.87, 51.14, 40.12, 24.67. ESI-MS *m/z*: 443 [M+H]^+^. HRMS [M+H]^+^ Calcd for C_27_H_27_FN_4_O: 443.2169, found: 443.2180.

**Synthesis of ethyl 3-amino-3-phenylpropanoate (18).**

A similar procedure to the one described by Gábor Tasnádi *et al* was followed with some modifications. EtOH (100 mL) was added dropwise in SOCl_2_ (5 mL), with the temperature being kept under -10 ^o^C. To this solution, compound **4a** (8.30g, 50 mmol) was added. The mixture was stirred at 0 ^o^C for 30 min, and then at room temperature for 3 h, and finally heated at reflux for 1 h. The solvent was evaporated off and the resulting **18.**HCl was recrystallized from EtOH and Et_2_O. Treatment of **18.**HCl with aqueous KOH resulted in the free product **18** as yellow oils (7.91g, 81%). The ^1^H-NMR data are in accordance with those reported in the literature ^[8]^. ESI-MS *m/z*: 194 [M+H]^+^.

**Synthesis of ethyl 3-(tert-butoxycarbonylamino)-3-phenylpropanoate (19).**

Di-tert-butyl pyrocarbonate (Boc_2_O, 2.41g, 11 mmol) was added in portions to the stirred solution of compound **18** (1.93g, 10 mmol) in dioxane (20 ml) and 10% aqueous NaOH solution (15 ml) at 0 ^o^C, then at room temperature for 12 h. The solution was extracted with EtOAc (3×30 mL). The combined organic layer was washed with brine (2×10 mL), dried (Na_2_SO_4_), filtered, and concentrated in vacuo to get product **19** as white solid (2.51g, 84%). m.p.: 59-61 ^o^C, ^1^H-NMR (500 MHz, CDCl_3_): *δ*: 7.36-7.29(m, 5H, Ar-H), 5.24-5.19(m, 1H), 4.15-4.12(m, 2H), 4.03-2.96(m, 2H), 1.41(s, 9H, 3×CH_3_), 1.35-1.33(m, 3H). ESI-MS *m/z*: 294 [M+H]^+^.

**Synthesis of tert-butyl 3-oxo-1-phenylpropylcarbamate (20).**

To a solution of compound **19** (1.47g, 5 mmol) in CH_2_Cl_2_ (10 mL) cooled to -78 °C, DIBAL-H (8.0 mL of 25% W/W in toluene, 10.0 mmol) was added dropwise in 0.5 h. The mixture was maintained at -78 °C for 2.5 h, and then quenched by the addition of MeOH (2 mL) and H_2_O (1 mL). After that, the reaction was allowed to warm to room temperature. The mixture was filtered and the filtrate was dried (Na_2_SO_4_), concentrated in vacuo. The residue was purified by column chromatography (petroleum ether : ethyl acetate = 1 : 1) to afford the product **20** as white solid (0.72g, 58%). m.p.: 83-87 ^o^C, ^1^H-NMR (500 MHz, CDCl_3_): *δ*: 9.75(s, 1H, CHO), 7.37-7.28(m, 5H, Ar-H), 5.20-5.15(m, 1H), 3.00-2.89(m, 2H), 1.42(s, 9H, 3×CH_3_). ESI-MS *m/z*: 250 [M+H]^+^.

The compound **21** were prepared from compounds **20** and **8c** by a method similar to that described for **9a-h**.

***Tert*-butyl 3-(4-(4-cyanophenyl)piperazin-1-yl)-1-phenylpropylcarbamate (21).** Pale yellow solid (40%). m.p.: 128-131 ^o^C. ^1^H-NMR (500 MHz, CDCl_3_): *δ*: 7.49(d, *J* = 8.5 Hz, 2H, Ar-H), 7.32(t, *J* = 8.5 Hz, 2H, Ar-H), 7.28-7.25(m, 3H, Ar-H), 6.85(d, *J* = 9.0 Hz, 2H, Ar-H), 6.05-6.02(m, 1H), 3.38-3.29(m, 4H, piperazinyl-H), 2.62-2.52(m, 4H, piperazinyl-H), 2.43-2.29(m, 2H), 2.10-2.03(m, 2H), 1.41(s, 9H, 3×CH_3_). ESI-MS *m/z*: 421 [M+H]^+^.

**General method for synthesis of compounds (23a-h).**

To a stirred solution of compound **21** (0.42g, 1 mmol) in EtOAc (2 mL) was added 6 N aqueous HCl (8 mL), and the mixture was stirred at room temperature for 3 h. The mixture was diluted with 10% aqueous NaOH solution (15 mL), and the organic layer was separated. The aqueous layer was extracted with EtOAc (3×25 mL), and the combined organic layer was washed with brine (2×10 mL), dried with Na_2_SO_4_, filtered, and concentrated in vacuo to get the product **22**, which was used directly without any purification.

A mixture of compound **22** (32 mg, 0.1 mmol), the appropriate carboxylic acid (0.1 mmol), and 1-ethyl-3-(3-dimethylaminopropyl)-carbodiimide hydrochloride (EDC.HCl) (22 mg, 0.12 mmol) in CH_2_Cl_2_ (5 mL) was stirred at room temperature for 6h. The mixture was evaporated in vacuo and purified by column chromatography on silica gel (petroleum ether : ethyl acetate = 1 : 4) to afford the compounds **23a-h**.

***N*-(3-(4-(4-Cyanophenyl)piperazin-1-yl)-1-phenylpropyl)benzamide (23a).** Pale yellow solid (63%). m.p.: 152-155 ^o^C. ^1^H-NMR (500 MHz, CDCl_3_): *δ*: 8.68(d, 1H, *J* = 7.0 Hz, NH), 7.83-6.82(m, 14H, Ar-H), 5.38-5.34(m, 1H), 3.27-3.14(m, 4H, piperazinyl-H), 2.78-2.64(m, 4H, piperazinyl-H), 2.62-2.59(m, 1H), 2.50-2.45(m, 1H), 2.31-2.24(m, 2H). ^13^C-NMR (125 MHz, CDCl_3_): *δ*: 166.80, 153.21, 141.88, 134.73, 133.56, 131.55, 128.70, 128.52, 127.28, 127.09, 126.29, 119.86, 114.39, 100.94, 54.97, 53.48, 52.87, 47.24, 31.73. ESI-MS *m/z*: 425 [M+H]^+^. HRMS [M+H]^+^ Calcd for C_27_H_28_N_4_O: 425.2263, found: 425.2245.

**4-Chloro-*N*-(3-(4-(4-cyanophenyl)piperazin-1-yl)-1-phenylpropyl)benzamide (23b).** Yellow solid (60%). m.p.: 143-145 ^o^C. ^1^H-NMR (500 MHz, CDCl_3_): *δ*: 8.59(d, 1H, *J* = 6.5 Hz, NH), 7.86-6.82(m, 13H, Ar-H), 5.35-5.31(m, 1H), 3.37-3.29(m, 4H, piperazinyl-H), 2.69-2.54(m, 4H, piperazinyl-H), 2.52-2.45(m, 2H), 2.30-2.21(m, 1H), 2.08-2.03(m, 1H). ^13^C-NMR (125 MHz, CDCl_3_): *δ*: 165.82, 153.15, 141.79, 137.79, 133.59, 133.04, 128.76, 128.74, 128.56, 127.37, 126.25, 119.84, 114.43, 101.01, 54.93, 53.58, 52.81, 47.20, 31.77. ESI-MS *m/z*: 459 [M+H]^+^. HRMS [M+H]^+^ Calcd for C_27_H_27_ClN_4_O: 459.1873, found: 459.1865.

***N*-(3-(4-(4-Cyanophenyl)piperazin-1-yl)-1-phenylpropyl)-4-(trifluoromethyl)benzamide (23c).** Yellow solid (68%). m.p.: 158-160 ^o^C. ^1^H-NMR (500 MHz, CDCl_3_): *δ*: 8.68(d, 1H, *J* = 6.0 Hz, NH), 7.81-6.82(m, 13H, Ar-H), 5.37-5.32(m, 1H), 3.39-3.26(m, 4H, piperazinyl-H), 2.65-2.53(m, 4H, piperazinyl-H), 2.51-2.44(m, 2H), 2.27-2.20(m, 1H), 2.10-2.04(m, 1H). ESI-MS *m/z*: 493 [M+H]^+^. HRMS [M+H]^+^ Calcd for C_28_H_27_F_3_N_4_O: 493.2137, found: 493.2120.

***N*-(3-(4-(4-Cyanophenyl)piperazin-1-yl)-1-phenylpropyl)-4-isopropylbenzamide (23d).** Yellow solid (45%). m.p.: 155-158 ^o^C. ^1^H-NMR (500 MHz, CDCl_3_): *δ*: 8.44(d, 1H, *J* = 7.0 Hz, NH), 7.87-6.86(m, 13H, Ar-H), 5.39-5.35(m, 1H), 3.40-3.27(m, 4H, piperazinyl-H), 2.70-2.55(m, 4H, piperazinyl-H), 2.69-2.50(m, 2H), 2.33-2.30(m, 1H), 2.26-2.20(m, 2H), 1.55(d, *J* = 5.0 Hz, 3H, CH_3_), 1.53(d, *J* = 5.0 Hz, 3H, CH_3_). ESI-MS *m/z*: 467 [M+H]^+^. HRMS [M+H]^+^ Calcd for C_30_H_34_N_4_O: 467.2733, found: 467.2720.

**1-Acetyl-*N*-(3-(4-(4-cyanophenyl)piperazin-1-yl)-1-phenylpropyl)piperidine-4-carboxamide (23e).** Yellow solid (45%). m.p.: 100-105 ^o^C. ^1^H-NMR (500 MHz, CDCl_3_): *δ*: 7.51-7.28(m, 6H, Ar-H), 7.13-6.86(m, 3H, Ar-H), 5.17-5.13(m, 1H), 3.39-3.30(m, 4H), 2.64-2.54(m, 4H), 2.45-2.33(m, 2H), 2.10-2.02(m, 2H), 2.00(s, 3H, CH_3_), 1.97-1.58(m, 9H). ESI-MS *m/z*: 474 [M+H]^+^. HRMS [M+H]^+^ Calcd for C_28_H_35_N_5_O_2_: 474.2791, found: 474.2780.

**1-Benzoyl-*N*-(3-(4-(4-cyanophenyl)piperazin-1-yl)-1-phenylpropyl)piperidine-4-carboxamide (23f).** Yellow solid (49%). m.p.: 121-124 ^o^C. ^1^H-NMR (500 MHz, CDCl_3_): *δ*: 7.51-6.85(m, 14H, Ar-H), 5.14-5.10(m, 1H), 4.66-4.61(m, 1H), 3.81-3.69(m, 2H), 3.38-3.25(m, 4H), 3.10-2.82(m, 2H), 2.63-2.55(m, 4H), 2.46-2.37(m, 4H), 2.20-2.06(m, 4H). ESI-MS *m/z*: 536 [M+H]^+^. HRMS [M+H]^+^ Calcd for C_33_H_37_N_5_O_2_: 536.2947, found: 536.2964.

***N*-(3-(4-(4-Cyanophenyl)piperazin-1-yl)-1-phenylpropyl)furan-2-carboxamide (23g).** Yellow solid (59%). m.p.: 132-136 ^o^C. ^1^H-NMR (500 MHz, CDCl_3_): *δ*: 8.68(d, 1H, *J* = 7.5 Hz, NH), 7.51-6.45(m, 12H, Ar-H), 5.35-5.33(m, 1H), 3.40-3.28(m, 4H, piperazinyl-H), 2.70-2.56(m, 4H, piperazinyl-H), 2.54-2.47(m, 2H), 2.37-2.34(m, 1H), 2.22-2.18(m, 1H). ^13^C-NMR (125 MHz, CDCl_3_): *δ*: 157.76, 153.40, 148.53, 143.34, 141.59, 133.59, 128.65, 127.24, 126.31, 119.91, 114.57, 114.27, 112.35, 100.72, 54.48, 52.79, 52.58, 47.26, 31.71. ESI-MS *m/z*: 415 [M+H]^+^. HRMS [M+H]^+^ Calcd for C_25_H_26_N_4_O_2_: 415.2056, found: 415.2078.

***N*-(3-(4-(4-Cyanophenyl)piperazin-1-yl)-1-phenylpropyl)cyclohexanecarboxamide (23h).** Yellow solid (43%). m.p.: 93-96 ^o^C. ^1^H-NMR (500 MHz, CDCl_3_): *δ*: 7.53-6.87(m, 9H, Ar-H), 5.15-5.12(m, 1H), 3.39-3.32(m, 4H, piperazinyl-H), 2.65-2.56(m, 4H, piperazinyl-H), 2.44-2.34(m, 2H), 2.21-2.16(m, 2H), 1.77-1.44(m, 11H). ESI-MS *m/z*: 431 [M+H]^+^. HRMS [M+H]^+^ Calcd for C_27_H_34_N_4_O: 431.2733, found: 431.2749.
